# Feasibility of designing, manufacturing and delivering 3D printed ankle-foot orthoses: a systematic review

**DOI:** 10.1186/s13047-019-0321-6

**Published:** 2019-02-07

**Authors:** Elizabeth Wojciechowski, Angela Y. Chang, Daniel Balassone, Jacqueline Ford, Tegan L. Cheng, David Little, Manoj P. Menezes, Sean Hogan, Joshua Burns

**Affiliations:** 1 The University of Sydney, Sydney, New South Wales Australia; 2 Paediatric Gait Analysis Service of NSW, The Children’s Hospital at Westmead, Cnr Hawkesbury Road and Hainsworth Street, Locked Bag 4001, Westmead, NSW 2145 Australia

**Keywords:** 3D printing, Additive manufacturing, Ankle foot orthoses, AFO

## Abstract

**Background:**

Ankle-foot orthoses (AFO) are prescribed to manage difficulty walking due to foot drop, bony foot deformities and poor balance. Traditional AFOs are handmade using thermoplastic vacuum forming which provides limited design options, is labour-intensive and associated with long wait times. 3D printing has the potential to transform AFO production and health service delivery. The aim of this systematic review was to determine the feasibility of designing, manufacturing and delivering customised 3D printed AFOs by evaluating the biomechanical outcomes, mechanical properties and fit of 3D printed compared to traditionally manufactured AFOs.

**Method:**

Electronic databases were searched from January 1985 to June 2018 according to terms related to 3D printing and AFOs. Studies of any design from healthy or pathological populations of any age were eligible for inclusion. Studies must have investigated the effect of customised 3D printed AFOs using any 3D printing technique on outcomes related to walking ability, biomechanical function, mechanical properties, patient comfort, pain and disability. Any other orthotic type or AFOs without a 3D printed calf and foot section were excluded. The quality of evidence was assessed using the GRADE process.

**Results:**

Eleven studies met the eligibility criteria evaluating 3D printed AFOs in healthy adults, and adults and children with unilateral foot drop from a variety of conditions. 3D printing was used to replicate traditional AFOs and develop novel designs to optimise the stiffness properties or reduce the weight and improve the ease of use of the AFO. 3D printed custom AFOs were found to be comparable to traditional custom AFOs and prefabricated AFOs in terms of temporal-spatial parameters. The mechanical stiffness and energy dissipation of 3D printed AFOs were found to be similar to prefabricated carbon-fibre AFOs. However, the sample sizes were small (*n* = 1 to 8) and study quality was generally low.

**Conclusion:**

The biomechanical effects and mechanical properties of 3D printed AFOs were comparable to traditionally manufactured AFOs. Developing novel AFO designs using 3D printing has many potential benefits including stiffness and weight optimisation to improve biomechanical function and comfort.

**Electronic supplementary material:**

The online version of this article (10.1186/s13047-019-0321-6) contains supplementary material, which is available to authorized users.

## Background

Ankle-foot orthoses (AFO) are externally worn medical devices that support the foot, ankle and lower leg to manage lower limb impairments such as foot drop, bony foot deformities and poor balance. They are routinely prescribed to improve the walking ability of children and adults with neurological disorders such as cerebral palsy, Charcot-Marie-Tooth disease, cerebrovascular accident (stroke) and multiple sclerosis [1]. Customised AFOs are often prescribed to prevent trips and falls resulting from foot drop, alleviate chronic pain associated with joint deformity, and control the ground reaction force during the stance phase of gait to reduce fatigue. Many users of AFOs experience poor fit, pain and discomfort, dislike the aesthetics of the devices and are limited by the choice of design and associated footwear [1–3]. In particular children and adolescents, females and people living alone are reported to be the most dissatisfied users of AFOs [1]. Hence, many children and adults with musculoskeletal and neuromuscular disorders don’t wear their prescribed AFOs and instead utilise compensatory strategies during gait despite these being physiologically inefficient and potentially injurious [4]. Therefore, many users only choose to wear their devices when their condition becomes severe even though earlier AFO use might have significant clinical benefits [4].

AFOs are usually handmade from plaster of Paris casting of the patient’s lower legs [5]. Once set, this negative impression is removed and filled with liquid plaster to form a positive model. The positive model is then modified through manual addition or removal of plaster, followed by thermoplastic vacuum forming over the positive model with polypropylene. Removal of undesired or excess polypropylene and smoothing occur prior to patient fitting. This traditional approach is labour intensive, provides limited design options, can be costly and often associated with long wait times [6]. 3D printing is a manufacturing method whereby materials are joined, layer by layer, to fabricate an object from a digital source. It has the potential to eliminate several steps associated with traditional methods of AFO manufacture [6]. 3D printing enables design freedom by facilitating deviation from traditional design paradigms and hence allows the development of patient-specific AFOs. These AFOs can be optimised to individual biomechanical requirements to provide improved function, better fit and enhanced aesthetics. Novel patient-specific 3D printed AFOs are likely to have a dramatic effect on patient satisfaction, adherence to AFO usage and overall health related outcomes. The aim of this systematic review of the literature was to investigate the feasibility of 3D printing for the design, manufacture and delivery of AFOs by evaluating biomechanical effects, mechanical properties and fit of 3D printed compared to traditionally manufactured AFOs.

## Method

The systematic review protocol was developed in accordance with the Preferred Reporting Items for Systematic Reviews and Meta-Analyses (PRISMA) statement [7].

### Search strategy

Electronic database searches were performed in June 2018 in MEDLINE (via OvidSP), EMBASE, AMED (via OvidSP), CINAHL (via EBSCO), Web of Science, Scopus, Cochrane and ProQuest Central according to search terms related to 3D printing (3D print*, three dimensional print*, additive manufactur*, rapid prototype*, additive fabricat*, additive process*, additive technique*, freeform fabricat*, selective laser sinter*, sterolithograpy, fusion deposition model*, laminated object manufactur*, layer manufactur*) combined with terms related to AFOs (ankle foot ortho*, AFO, static ankle foot ortho*, fixed ankle foot ortho*, solid ankle foot ortho*, ground reaction ankle foot ortho*, floor reaction ankle foot ortho*, dynamic ankle foot ortho*, hinged ankle foot ortho*, articulation ankle foot ortho*, passive ankle foot ortho*). 3D printing commenced in the mid-1980s [8], therefore searches were from January 1985. The search strategy was developed for MEDLINE and modified for use in other databases (Additional file 1).

### Eligibility criteria

Studies of human participants of any sample size were eligible, and there were no age, sex, cultural or ethnicity restrictions. Participants were either from healthy or any clinical populations. Studies must have investigated the effect of customised 3D printed AFOs using any 3D printing technique [stereolithography (SLA), fused deposition modelling (FDM), laminated object manufacturing (LOM), selective laser sintering (SLS), selective laser melting (SLM)] on outcomes related to walking ability or biomechanical function, mechanical properties, patient comfort, pain and disability. Any other orthotic type (e.g. below ankle, University of California Berkeley Laboratory orthoses, supermalleolar, knee-ankle-foot orthoses) or orthoses not used for walking (e.g. night use) or any other manufacturing technique (e.g. CAD/CAM where the machine carves a block to form an orthosis based on digital model milling) other than 3D printing, or AFOs where the calf and foot section were not 3D printed were excluded. All study designs were included, except for narrative and systematic reviews. Although reviews were excluded, the reference lists were examined for any additional relevant references. Unpublished data and data from studies with no full-length publication were excluded.

### Study selection, data extraction and study quality

Following the deletion of duplicates, titles and abstracts from the search results were screened by two authors (E.W. and D.B.) using the predetermined eligibility criteria. Full-text articles were retrieved for the remaining articles and independently reviewed by two authors (E.W. and D. B.) for inclusion. Data extraction and evaluation was then completed on the remaining articles by two authors (E.W. and J. F.) independently. Where there was disagreement at any stage an additional reviewer (J.B.) was consulted. Data extraction included study design, sample size, participant characteristics, orthotic details and outcome measures.

The Oxford Centre for Evidence-Based Medicine 2011 Levels of Evidence (OCEBM Levels) was used to determine a level of evidence for each study based on the design [9]. The American Academy for Cerebral Palsy and Developmental Medicine (AACPDM) conduct rating was used to critique the individual studies during the data extraction data process [10]. The overall quality of evidence was then assessed using the Grading of Recommendations Assessment, Development and Evaluation (GRADE) process (GRADEpro software) [11, 12].

## Results

### Description of included studies

**Fig. 1:**
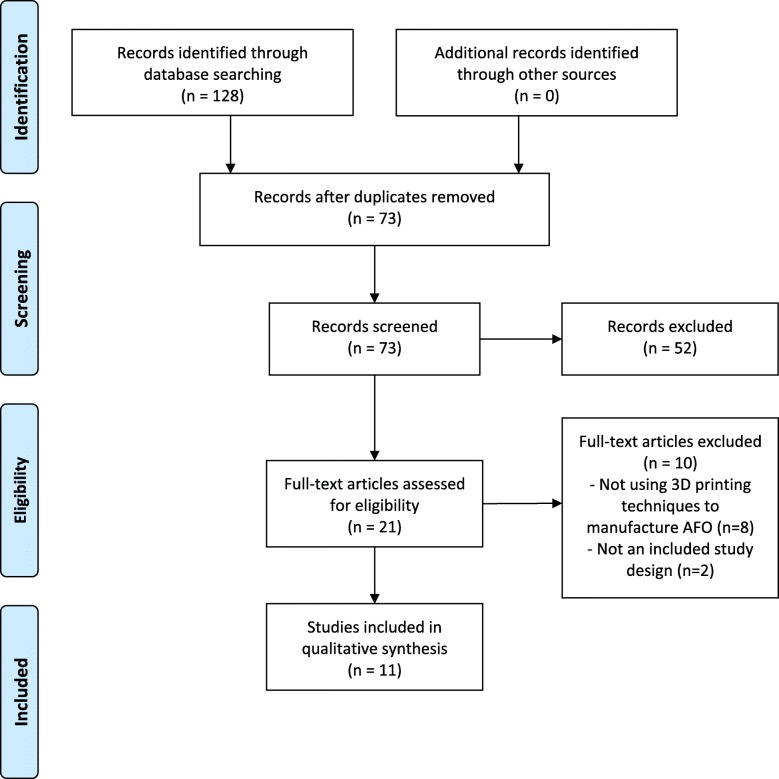
Flow diagram of the search history and selection process

The initial electronic database search resulted in a total of 128 articles, leaving 73 articles after the removal of duplicates. No additional articles were identified following a hand search of reference lists. Upon completion of the title and abstract screening, 21 were selected for possible inclusion in the review and full-text articles were retrieved. Following the screening of the full text, 11 studies met the inclusion criteria and were included in this review [13–23]. A flow diagram of the search history and selection process is shown in Fig. 1. The 11 studies included one case-control trial [13], eight case studies [14–18, 21–23], one prototyping study [19] and one finite element analysis study [20]. A description of the AFO details included in the studies can be found in Table 1.

**Table 1:** Participant characteristics, 3D printed orthotic details and outcomes of includes studies

Reference	AACPDM level of evidence & conduct rating	Participants’ Characteristics		Orthotic Details		Outcomes and Results		
	Study Design	N	Condition	Intervention vs control condition	3D printing method and material	Outcomes	Main Results and Authors conclusions	OCEBM level
Aydin et al., 2018 [20]	V (1/7) Computing analysis and prototyping	1	Healthy participant	Customised FDM AFO vs no control	FDM ABS	FEA: Material displacement	Material displacement of the AFO model using mechanical properties from 3D tested specimens was higher compared to the using mechanical properties from supplied with the FEA software.	5
Deckers et al., 2018 [21]	V (1/7) Case-studies	7	Trauma, neuro-muscular disorder and cerebral palsy (3 children, 4 adults)	Customised SLS AFO with a 3 mm thick calf and foot section connected with 2 carbon fibre rods (6 weeks) vs traditionally manufactured AFOs (6 weeks)	SLS Polyamide 12 (PA12)	Observation after 6-week trial	No noticeable failure or wear with the traditionally manufactured AFOs after 6 weeks. 5/7 SLS AFO broke during the 6-week period, 1 SLS AFO showed signs of cracking and 1 did not fail.	4
Cha et al., 2017 [22]	V (1/7) Case study	1	Right side foot drop after embolectomy (female, 68 yrs)	Novel customised SLS AFO vs traditional polypropylene AFO (altered wear over 2 months)	FDM Polyurethane	Durability test of 300,000 cycles QUEST after 2 months 3DGA: temporal spatial parameters, ankle kinematics	No crack, shape or stiffness change following the durability test. The participant was more satisfied with 3D printed AFO in terms of weight and ease of use. Temporal spatial parameters were similar between AFOs however ankle dorsiflexion in swing was less with the 3D printed AFO compared to the traditional AFO.	4
Choi et al., 2017 [23]	IV (3/7) Case-studies	8	Healthy participants (4 male, 4 female 25.3 SD 4.5 yrs)	Customised articulated FDM AFO with a metal hinged joint and 2 elastic polymer bands at 4 levels of stiffness and no resistance vs no control	FDM PLA	3DGA: kinematics Ultrasound Musculoskeletal modelling	Increasing AFO stiffness increased peak ankle dorsiflexion moment and decreased peak knee extension and peak ankle dorsiflexion. The method may assist AFO design and prescription to improve gait.	4
Creylman, et al., 2013 [13]	IV (3/7) Case-control	8	Unilateral drop foot due to dorsiflexor weakness from multiple conditions (male 46.6 yrs. SD 12.5)	Customised SLS AFO vs traditionally manufactured polypropylene AFO vs barefoot	SLS Nylon 12 (PA 2201)	3DGA: temporal spatial parameters and kinematics.	No statistically significant differences between the traditionally manufactured AFO and of SLS AFO in terms of temporal spatial gait parameters, ankle angle at initial contact and maximum ankle plantarflexion during swing. Significant differences were noted in ankle range of motion. Authors attribute this to differences in material stiffness.	4
Faustini, et al., 2008 [14]	V (1/7) Case-study	1	Post-Polio Syndrome (male 66 yrs)	SLS PD-AFO vs Dynamic Brace CF-AFO	SLS Nylon 11 (Rilsan D80), Nylon 12 (DuraForm PA) and glass-filled Nylon 12 (DuraForm GF)	Rotational stiffness, energy dissipation & destructive testing.	Nylon 11 exhibited the least amount of mechanical damping and was the only material to withstand the destructive testing	4
Mavroidis, et al., 2011 [15]	V (2/7) Case-study	1	Healthy participant	Customised SLA AFO (rigid & flexible) vs prefabricated injection moulded polypropylene AFO vs shod only	SLA Accura 40 resin and DSM Somos 9120 Epoxy Photopolymer	3DGA: temporal spatial parameters, kinematics and kinetics. Patient perceived fit.	3D printed AFOs provided good fit to the participant’s anatomy and were comparably to the prefabricated AFO during gait	4
Schrank and Stanhope., 2011 [16]	V (0/7) Case-studies	2	Healthy participants (male 48 yrs.; female 21 yrs)	4 half scale PD-AFO and two full-scale PD-AFO vs no control	SLS Nylon (DuraForm EX Natural Plastic)	Dimensional accuracy. Patient perceived fit.	Dimension discrepancies were well under a 2 mm tolerance for the four half-scale orthoses. Subjective evaluations of the full-scale PD-AFOs following use in gait were positive	4
Schrank, et al., 2013 [15]	V (0/7) Case-studies	2	Healthy participants (male 25 yrs.; female 24 yrs)	2 sets of stiffness tuned PD-AFOs vs no control	FDM medical-grade polycarbonate (PC-ISO).	Dimensional accuracy, manufacturing precision and bending stiffness prediction accuracy.	The virtual functional prototyping had excellent dimensional accuracy, good manufacturing precision and strong predication accuracy with the derived modulus	4
Telfer, et al., 2012 [18]	V (1/7) Case-study	1	Healthy participant (male 29 yrs)	Customised SLS AFO at two different stiffness levels vs shod only	SLS Nylon-12 (PA2200)	3DGA: kinematics and kinetics	The AFO had distinct effects on ankle kinematics which could be varied by adjusting the stiffness level of the device	4
Walburn, et al., 2016 [19]	V (0/7) Prototyping	0	None	A novel segmented 3D printed and CFRP AFO vs no control	FDM ABS	Linear stiffness coefficient	A novel segmented 3D printed and CFRP AFO has been proposed.	5

*AACPDM* American Academy for Cerebral Palsy and Developmental Medicine, *OCEBM* Oxford Centre for Evidence-Based Medicine Levels of Evidence, *AFO* Ankle-foot orthoses, *PD-AFO* Passive-dynamic ankle foot orthoses, *CFRP* carbon fibre reinforce spring, *SLS* Selective laser sintering, *SLA* Stereolithography, *FDM* Fused deposition modelling, *3DGA* Three-dimensional gait analysis, *FEA* Finite element analysis, *QUEST* Quebec User Evaluation of Satisfaction with Assistive Technology

### Quality of included studies

Nine of the studies were OCEBM level 4 and two level 5 [Table 1]. The conduct rating of the studies using the AACPDM was moderate for two studies [13, 23] and weak for all other studies (Table 1 and Additional file 2). The outcomes assessed using the GRADE process included walking ability, patient satisfaction, patient-perceived comfort, bending stiffness, energy dissipation, destructive testing, dimensional accuracy between the CAD model and printed AFO, durability and material displacement [Table 2]. The GRADE overall quality evidence for all these outcomes was considered very low.

**Table 2:** GRADE evidence profile

Quality assessment							№ of patients		Effect		Quality	Importance
№ of studies	Study design	Risk of bias	Inconsistency	Indirectness	Imprecision	Other considerations	AFOs manufactured using 3D printing techniques	Traditionally manufactured AFOs	Relative (95% CI)	Absolute (95% CI)		
Walking ability (assessed by 3D gait analysis)												
5	observational studies ^1,2,3,4,5^	serious ^a,b,c^	not serious	serious ^1,5,6,7 a,b^	serious ^d^	none	20	11	–	–	⨁◯◯◯ VERY LOW	IMPORTANT
Patient perceived comfort (assessed by interview)												
2	observational studies ^2,8^	very serious ^e^	not serious	serious ^a^	serious ^d^	none	Interview after use of AFO during gait				⨁◯◯◯ VERY LOW	IMPORTANT
Patient satisfaction (assessed with the QUEST)												
2	observational studies ^4^	very serious ^f^	not serious	not serious	serious ^d^	none	1	1	–	–	⨁◯◯◯ VERY LOW	IMPORTANT
Bending stiffness (assessed by bench testing using custom stiffness testing device)												
3	observational studies ^6,9,10^	serious ^a,c,g^	not serious	serious ^a^	serious ^d^	none	3	1	–	–	⨁◯◯◯ VERY LOW	IMPORTANT
Energy Dissipation (assessed by bending testing and analysing the resulting acceleration-time trajectory)												
1	observational studies ^6^	not serious	not serious	serious ^a^	serious ^d^	none	1	1	–	–	⨁◯◯◯ VERY LOW	IMPORTANT
Destructive Testing (assessed by benching testing using a hydraulic axial load cell)												
1	observational studies ^4^	not serious	not serious	serious ^a^	serious ^d^	none	1	1	–	–	⨁◯◯◯ VERY LOW	IMPORTANT
Dimensional accuracy between CAD model and printed AFO (assessed by the FaroArm, fit with a 3 mm spherical tip)												
1	observational studies ^8^	serious ^h^	not serious	serious ^a^	serious ^d^	none	2	–	–	–	⨁◯◯◯ VERY LOW	IMPORTANT
Durability (assessed by mechanical stress test of 300,000 cycles)												
1	observational studies ^4^	serious ^a^	not serious	serious ^a^	serious ^d^	none	2	–	–	–	⨁◯◯◯ VERY LOW	IMPORTANT
Durability (follow up: 6 weeks; assessed by observation)												
1	observational studies ^11^	serious ^d,e^	not serious	not serious	serious ^d^	none	7	7	–	–	⨁◯◯◯ VERY LOW	IMPORTANT
Material displacement (assessed by finite element analysis)												
1	observational studies ^7^	serious ^h,i^	not serious	serious ^a^	serious ^d^	none	1	–	–	–	⨁◯◯◯ VERY LOW	IMPORTANT

*CI* Confidence interval

^1^Telfer, et al., 2012, ^2^Mavroidis, et al., 2011, ^3^Creylman, et al., 2013, ^4^Cha, et al., 2017, ^5^Choi et al. 2017., ^6^Faustini, et al., 2008, ^7^Aydin et al. 2018 ^8^Schrank and Stanhope, 2011, ^9^Schrank, et al. 2013, ^10^Walbran, et al., 2016, ^12^Deckers, et al., 2018 Explanations: ^a^ Not all studies compared to traditionally manufactured AFOs, ^b^ Differences in study populations, ^c^ Differences in type of AFO assessed, ^d^ Participants / number of AFOs assessed low, ^e^ No quantitative assessment, ^f^ No blinding of AFOs, ^g^ Method used to assess outcome different across studies, ^h^ Influence of oblique build orientations and positions and different sized and shaped orthoses not tested, ^i^ Number of samples tested low

### Participant characteristics

Out of the 11 studies a total of 32 adult participants participated (17 male, 6 female, 9 sex not specified). The average age was 39 ± 17 years and ranged from 21 to 68 years, however three studies did not report the age of the participants [19–21]. Sample sizes were *n* = 1 [14, 15, 18, 20, 22], *n* = 2 [16, 17], *n* = 7 [21] and *n* = 8 [13, 23]. Four out of the 11 studies were conducted on patient populations including unilateral foot drop due to dorsiflexor weakness from stroke, cerebral palsy, L5 hernia, carbon monoxide intoxication and mechanical trauma [13], post-polio syndrome [14], trauma and cerebral palsy [21] and an embolectomy [22]. Six studies recruited healthy participants [15–18, 20, 23] and one study did not report or evaluate the AFO in any participants (bench testing only) [19].

### Orthotic details

#### Type of AFO

Ten of the 11 studies evaluated the feasibility of using 3D printing to produce a type of dynamic passive AFO, which relies on the material properties and physical features to establish functional characteristics such as bending or rotational stiffness. Specifically, two studies replicated a posterior leaf spring AFO [13, 15], one study replicated the geometry and design characteristics of a prefabricated carbon-fibre AFO (Dynamic Brace, Dynamic Bracing Solutions, Inc., San Diego, CA) [14] and six studies developed novel AFOs [16–19, 21, 22]. The novel designs included the development of a parameterised AFO using computer aided modelling [16, 17]. One study integrated a 3D printed component with off-the-shelf bearings and gas springs to produce an adjustable stiffness AFO [18]. Another study manufactured a segmented AFO consisting of a 3D printed calf section and foot section and a central interchangeable carbon fibre spring [19]. Similarly, another study integrated a commercially available metal hinge also capable of adjusting the stiffness of the AFO into a 3D printed articulated AFO [23]. Other designs also included an AFO with a 3D printed 3 mm calf and foot section connected with two carbon fibre rods [21] and a 3D printed device supporting the ankle and foot and secured with laces [24]. The only study that didn’t produce a dynamic passive AFO used 3D printing to produce a rigid (solid) AFO, however no testing was performed on the manufactured AFO [20].

#### Comparative AFO

Three studies compared 3D printed AFOs to traditionally manufactured custom AFOs [13, 21, 22]. One study compared 3D printed AFOs to the Dynamic Brace carbon fibre AFO [14] and one study made comparisons to prefabricated injection moulded AFOs [15]. Six studies did not compare 3D printed AFOs to any other traditionally dispensed AFOs [16–20, 23].

#### 3D printing method and material

Six studies used selective laser sintering (SLS) to produce AFOs [13, 14, 16, 18, 21, 22], one study used stereolithography (SLA) [15] and four studies used fused deposition modelling (FDM) [17, 19, 20, 25]. The types of material used for fabrication of the AFOs varied considerably. Three studies used Nylon 12 (PA2201, DuraForm PA, PA2200) [13, 14, 18]. A range of materials were used in the remaining studies including Rilsan D80 (Nylon 11) [14], DuraForm GF (Glass filled Nylon 12) [14], Accura 40 resin [15], DSM Somos 9120 Exposy Photopolymer [15], DuraFrom Ex [16], medical-grade polycarbonate [17], acrylonitrile butadiene styrene (ABS) [19, 20], polyamide 12 (PA12) [21], polyurethane [22] and polylactide (PLA) [23]. Two studies compared different types of 3D printing materials by printing the same AFO in more than one material, one study compared Rilsan D80, DuraForm PA and DuraForm GF [14] and one study compared Accura 40 resin and DSM Somos 9120 Epoxy Photopolymer [15].

### Outcomes

The reported outcomes included walking ability assessed using 3D gait analysis [13, 15, 18, 23, 26], patient satisfaction assessed using the Quebec User Evaluation of Satisfaction with Assistive Technology (QUEST) [22], patient-perceived comfort assessed by interview [15, 16], dimensional accuracy between CAD model and 3D printed AFO [16, 17] and mechanical properties of the AFO. The reported mechanical properties included stiffness [14, 17, 19], energy dissipation [14], destructive testing [14] and cyclic-fatigue loading [22] assessed using bench testing equipment and material displacement using finite element analysis (FEA) [20]. No studies reported pain or disability reduction as an outcome.

#### Walking ability

Five studies evaluated the effect of 3D printed AFOs on walking ability [13, 15, 18, 23, 26]. Creylman and colleagues [13], compared SLS AFOs and traditionally manufactured AFOs to barefoot walking. They found that use of either AFO showed significant benefit in terms of stride length and stance phase duration of the affected limb and ankle kinematics compared to barefoot walking. No statistically significant differences in temporal spatial parameters (stride duration, stride length and stance phase duration of both affected and unaffected limb), ankle angle at initial contact and maximum ankle plantarflexion during swing were found between the traditionally manufactured AFOs and SLS AFOs. However, significant differences were noted in ankle range of motion over the whole gait cycle between the traditionally manufactured AFOs and SLS AFOs, with the SLS AFO showing a smaller range of motion. The authors attributed these differences to differences in material stiffness between the AFOs. Similarly, Mavroidis and colleagues [15], found that a prefabricated AFO allowed more ankle range of motion compared to two SLA AFOs (rigid and flexible) possibly due to greater compliance of the polypropylene material. However, overall ankle kinematics and kinetics of the three AFOs were similar. In contrast, Telfer and colleagues [18], evaluated the effect of a SLS AFO set at two different stiffness levels in the sagittal plane to shoe only walking. The stiffness of the SLS AFO was controlled by adjusting pressure in gas springs. Significantly reduced plantarflexion during the early stance phase was observed between stiffness conditions, with the higher stiffness setting allowing the minimal amount of plantarflexion. They suggested that tailoring the stiffness of SLS AFOs may provide support to suit different activities in a way that traditional AFOs are unable to offer. Similarly, Choi and colleagues [23] describe a method of evaluating the impact of AFO stiffness on the Achilles tendon and gastrocnemius function using an articulated FDM AFO with a metal hinge and two elastic polymer bands. The AFO was set at no resistance and 4 levels of stiffness. Increased AFO stiffness resulted in increased peak ankle dorsiflexion moment and decreased peak knee extension and peak ankle dorsiflexion angles. The authors propose that this method may assist in AFO design and prescription to improve gait and function.

Cha and colleagues [22] also used 3D gait analysis to compare an FDM polyurethane AFO tightened with shoe laces to a traditionally manufactured rigid polypropylene AFO in one participant. They found that both AFOs similarly improved temporal spatial parameters compared to barefoot walking. However, ankle kinematic data showed that the traditional AFO was more effective in supporting ankle dorsiflexion during swing compared to the 3D printed AFO. The authors suggested that this 3D printed AFO needed to be designed in more dorsiflexion to compensate for stretching of the AFO during wear. However, this is likely due to the design of the 3D printed AFO which was developed to be more like a supramalleolar orthosis rather than an AFO as the device only surrounded the ankle and hindfoot and not the lower leg.

#### Patient-perceived comfort

Only one study used a patient satisfaction questionnaire (QUEST) [22] to compare a traditionally manufactured AFO to a 3D printed AFO. This study found that the participant was more satisfied with 3D printed AFOs in terms of weight and ease of use. The traditional AFO was difficult to wear due to the thickness. Two studies assessed patient-perceived comfort through interview. Participants reported greater comfort whilst wearing the 3D printed AFO [15] and positive feedback during gait after 1 h of walking [16]. However, comparisons were not made to traditionally manufactured AFOs and no quantitative assessment of patient comfort or fit was performed in these studies.

#### Dimensional accuracy between CAD model and 3D printed AFO

Two studies assessed the dimensional accuracy between the CAD model of the AFO and 3D printed AFOs by measuring 3D inter-dimple distances on the foot plate, strut and cuff of the 3D printed AFO using a FaroArm fit with a 3 mm spherical tip [16, 17]. Dimensional discrepancies were < 2 mm tolerances for four half-scaled AFOs [16]. However, the influence of build orientation, device size or shape were not tested.

#### Mechanical properties

Three studies evaluated the bending or rotational stiffness of 3D printed AFOs [14, 17, 19] using custom experimental stiffness testing devices. Two studies measured stiffness to validated finite element analysis (FEA) [14, 17]. Faustini and colleagues [14] measured rotational stiffness by clamping the footplate of the AFO against a vertical base and applying an increasing vertical force to the cuff and found that the stiffness measures of the SLS AFOs manufactured in Rilsan D80 (Nylon 11), DuraForm PA (Nylon 12) and DuraFrom GF (glass-filled Nylon 12) were within in 5% of the targeted carbon fibre AFO stiffness values. Similarly, Schrank and colleagues [17] measured bending stiffness using a custom experimental stiffness testing device and found that the stiffness predication accuracy of the FDM AFO printed in polycarbonate was strong (average 0.20+/− 0.14 Nm/deg).

FEA has also been used to determine the material displacement of an AFO computer model [20]. Aydin and colleagues [20] derived the mechanical properties from 3D printed test specimens manufactured with different infill densities. The material displacement of the AFO model was higher when mechanical properties were derived from test specimens compared to when mechanical properties were supplied from FEA software (SolidWorks 2016). This illustrates the need to use mechanical properties from 3D printed test specimens rather than default material properties.

In addition to measuring stiffness, Faustini and colleagues [14] also measured energy dissipation and performed destructive testing on the 3D printed AFOs. They established that the AFO fabricated with Rilsan D80 (Nylon 11) exhibited the least amount of mechanical damping and was the only material to withstand destructive testing compared to AFOs fabricated in DuraForm PA (Nylon 12) and DuraForm GF (glass-filled Nylon 12) [14].

The only study to conduct durability testing of a 3D printed AFO performed 300,000 cycles of mechanical stress testing and found there was no damage, shape or stiffness change of the AFO printed in polyurethane [22].

## Discussion

The main finding of this systematic review was that 3D printed AFOs were found to be comparable to traditionally manufactured custom AFOs, prefabricated AFOs and prefabricated carbon-fibre AFOs in terms of temporal spatial parameters, ankle kinematics, mechanical stiffness, and energy dissipation. However, only five of the 11 studies compared 3D printed AFOs to traditionally manufactured AFOs. Populations studied were primarily healthy adults and patients with foot drop. Only one study evaluated 3D printed AFOs in children. The sample sizes of the studies retrieved were small (*n* = 1 to 8) and study quality was generally low.

The studies retrieved evaluated the feasibility of 3D printing a posterior leaf spring AFO [13, 15], a carbon-fibre AFO [14], a rigid AFO [20] and various novel AFO designs [16–19, 21–23]. None of the studies assessed the feasibility of 3D printing a traditional hinged AFO with a plantarflexion stop. The novel AFO approaches maximised the design freedom of 3D printing to either optimise the stiffness properties of the AFO [16–19, 23] or reduced the weight and ease of use [22]. Telfer and colleagues [18] integrated 3D printed components (shank section, strut, foot section and slider) with off-the-shelf bearings and gas springs to produce an adjustable stiffness AFO [18]. The combination of off-the-shelf and 3D printed components allows the user to lower the stiffness and tailor the support provided depending on their current activity. Similarly, Schrank and colleagues [17] optimised the stiffness of 3D printed AFOs using CAD and FEA to tune and predict the properties of a parameterised AFO. Optimising AFO stiffness is likely to improve lower limb biomechanics such as ankle power at push off, make tasks such as ascending and descending stairs more efficient, and reduce trips, fatigue and pain. However, none of the novel AFOs were tested in specific patient populations or were designed with a particular patient group in mind.

Deckers and colleagues [21] and Cha and colleagues [22] were the only studies to test their novel AFO designs in patient populations and over time. Deckers and colleagues [21] tested an AFO design with a 3D printed calf and foot section connected by two carbon fibre rods in children and adolescents with pathologies from trauma, neuromuscular disorder (unspecified) and cerebral palsy over a 6-week period. However, only one out of the seven 3D printed AFOs did not break or fail during the 6-week period. This highlights the need for appropriate design and validation of devices in a safe laboratory environment to ensure that the basic performance of the novel device is demonstrated. Validation of novel designs could be achieved through bench testing, computer modelling and testing with patient populations in a controlled laboratory setting before any user validation in the natural environment over longer periods of time is performed.

Consideration should also be given to the age, gender, footwear and lifestyle of the user when designing and developing new devices. Out of the 11 studies retrieved, only one study collected a patient satisfaction questionnaire, two reported subjective feedback on comfort and fit of the device and none evaluated pain or disability reduction. Poor AFO satisfaction has been identified in children and adolescents, females and people living alone due to experiences with poor fit, pain and dislike of the aesthetics of the devices [1–3]. Aspects of the device that users want improved are the size, weight, adjustability, finish and durability of their AFOs [1]. Cha and colleagues [22] showed improvement in patient satisfaction in terms of weight and ease of use for their novel AFO design, however the device did not prevent foot drop as much as the traditionally manufactured AFO [22]. In the future, evaluation of novel 3D printed designs in specific groups, such as children, should explore comfort and fit, pain and disability as well as overall satisfaction and adherence and the biomechanical function of the device.

The 3D printing method and materials varied markedly between studies. Faustini and colleagues [14] were the only study reporting some of the mechanical properties of AFOs manufactured using SLS in more than one material (Nylon 11, Nylon 12 and glass-filled Nylon 12). They concluded that Nylon 11 was the optimum material in terms of mechanical damping and deformation. Only one study evaluated the durability of the AFO through cyclic loading tests [19] however no studies evaluated the lifespan of the AFO or performed long-term follow up of greater than two months. The 3D printing methods reported were SLS, SLA and FDM. SLA is a method of 3D printing that utilises one or more ultraviolet lasers to cure a liquid photopolymer resin. However, the structural, functional and aesthetic integrity of an SLA product may be compromised with prolonged UV exposure, where the material becomes brittle or discoloured [27, 28]. Therefore, SLA devices may require further material science studies or post-processing strategies to mitigate exposure to UV. SLS is similar to SLA however instead of using a laser to cure a liquid, a laser is used to trace and fuse or melt a powder substrate to fabricate an object, layer by layer. SLS manufactured AFOs also require lengthy ‘warm up’ and ‘cool down’ periods for optimal fabrication and safe removal. Indeed, one study reported a ‘cool down’ time of 6 h for the print bed to drop below 50^°^C prior to retrieval of the fabricated AFO [14]. FDM describes the process of depositing melted material through a heated extruder onto a platform. After the extruder has deposited a single cross-sectional layer of material the build platform is lowered to allow for printing of the next layer. Walbran and colleagues [19], used FDM to manufacture a segmented AFO. Segmentation of the calf and foot section into two parts allows optimal orientation for maximum strength during FDM manufacturing, whereas currently a single piece AFO cannot be orientated in a way where the strength is maximised in all directions [19]. Further research is required to determine the ideal printing material, printing method, print orientation and post processing for optimum durability and long-term usage.

## Conclusion

The use of 3D printing to manufacture AFOs seems to have many potential benefits over traditional methods, including the development of novel designs that optimise stiffness and energy dissipation, improve walking biomechanics, comfort and fit. The feasibility of using 3D printing to manufacture AFOs is dependent on the AFO design and printing method and therefore additional research is needed before 3D printed AFOs can be integrated into clinical practice. Further research is required to evaluate 3D printing AFOs in paediatric populations, and to determine the most appropriate printing technique and optimal materials to improve walking ability, patient satisfaction and long-term usage and durability.

## Additional files

Additional file 1: Search strategy for Medline, modified for other databases. (DOCX 26 kb)

Additional file 2: Conduct of group design studies. (DOCX 26 kb)
