# Supplementary material for: Feasibility of designing, manufacturing and delivering 3D printed ankle-foot orthoses: a systematic review
Source: J Foot Ankle Res. 2019 Feb 7;12:11. doi: 10.1186/s13047-019-0321-6 (PMC6367826; doi:10.1186/s13047-019-0321-6)
Supplement: Supplementary file 1 — Search strategy for Medline, modified for other databases. (DOCX 26 kb) [file 13047_2019_321_MOESM1_ESM.docx]

**Additional file 1.** Search strategy for Medline, modified for other databases.

| Ovid MEDLINE(R) <1946 to June Week 3 2018> | | |
| --- | --- | --- |
| **#** | **Search Statement** | **Results** |
| 1 | Printing, Three-Dimensional/ | 2056 |
| 2 | 3D print*.mp. | 1890 |
| 3 | three dimensional print*.mp. | 661 |
| 4 | additive manufactur*.mp. | 535 |
| 5 | rapid prototyp*.mp. | 1652 |
| 6 | additive fabricat*.mp. | 8 |
| 7 | additive process*.mp. | 50 |
| 8 | additive technique*.mp. | 15 |
| 9 | freeform fabricat*.mp. | 120 |
| 10 | solid freeform fabricat*.mp. | 102 |
| 11 | selective laser sinter*.mp. | 190 |
| 12 | sterolithography.mp. | 5 |
| 13 | fusion deposition model*.mp. | 2 |
| 14 | laminated object manufactur*.mp. | 7 |
| 15 | selective laser melt*.mp. | 166 |
| 16 | additive layer manufactur*.mp. | 5 |
| 17 | layer manufactur*.mp. | 20 |
| 18 | 1 or 2 or 3 or 4 or 5 or 6 or 7 or 8 or 9 or 10 or 11 or 12 or 13 or 14 or 15 or 16 or 17 | 4891 |
| 19 | ankle foot ortho*.mp. | 817 |
| 20 | AFO.mp. | 428 |
| 21 | ankle-foot ortho*.mp. | 817 |
| 22 | static ankle foot ortho*.mp. | 3 |
| 23 | static ankle-foot ortho*.mp. | 3 |
| 24 | fixed ankle foot ortho*.mp. | 5 |
| 25 | fixed ankle-foot ortho*.mp. | 5 |
| 26 | solid ankle foot ortho*.mp. | 21 |
| 27 | solid ankle-foot ortho*.mp. | 21 |
| 28 | ground reaction ankle foot orthos*.mp. | 2 |
| 29 | ground reaction ankle-foot orthos*.mp. | 2 |
| 30 | floor reaction ankle foot ortho*.mp. | 4 |
| 31 | floor reaction ankle-foot ortho*.mp. | 4 |
| 32 | dynamic ankle foot orthos*.mp. | 30 |
| 33 | dynamic ankle-foot orthos*.mp. | 30 |
| 34 | hinged ankle foot ortho*.mp. | 22 |
| 35 | hinged ankle-foot ortho*.mp. | 22 |
| 36 | articulating ankle foot ortho*.mp. | 1 |
| 37 | articulating ankle-foot ortho*.mp. | 1 |
| 38 | passive dynamic ankle foot ortho*.mp. | 10 |
| 39 | passive dynamic ankle-foot ortho*.mp. | 10 |
| 40 | passive-dynamic ankle-foot ortho*.mp. | 10 |
| 41 | 19 or 20 or 21 or 23 or 24 or 25 or 26 or 27 or 28 or 29 or 30 or 31 or 32 or 33 or 34 or 35 or 36 or 37 or 38 or 39 or 40 | 970 |
| 42 | 18 and 41 | 10 |
| 43 | limit 42 to yr="1985 -Current" | 10 |
