# Supplementary material for: Feasibility of designing, manufacturing and delivering 3D printed ankle-foot orthoses: a systematic review
Source: J Foot Ankle Res. 2019 Feb 7;12:11. doi: 10.1186/s13047-019-0321-6 (PMC6367826; doi:10.1186/s13047-019-0321-6)
Supplement: Supplementary file 2 — Conduct of group design studies. (DOCX 26 kb) [file 13047_2019_321_MOESM2_ESM.docx]

**Additional file 2.** Conduct of group design studies

| **Study** | **Level/ Quality** | **Conduct Questions*** | | | | | | |
| --- | --- | --- | --- | --- | --- | --- | --- | --- |
|  |  | **1** | **2** | **3** | **4** | **5** | **6** | **7** |
| Aydin et al., 2018 | V (1/7) | No | No | Yes | No | No | No | No |
| Deckers et al., 2017 | V (1/7) | Yes | No | No | No | No | No | No |
| Cha et al., 2017 | V (1/7) | No | No | Yes | No | No | No | No |
| Choi et al., 2017 | IV (3/7) | Yes | Yes | Yes | No | No | No | No |
| Creylman et al., 2013 | IV (3/7) | Yes | Yes | Yes | No | No | No | No |
| Faustini et al., 2008 | V (1/7) | No | Yes | No | No | No | No | No |
| Mavroidis et al., 2011 | V (2/7) | No | Yes | Yes | No | No | No | No |
| Schrank et al., 2011 | V (0/7) | No | No | No | No | No | No | No |
| Schrank et al., 2013 | V (0/7) | No | No | No | No | No | No | No |
| Telfer et al., 2012 | V (1/7) | No | No | Yes | No | No | No | No |
| Walburn et al., 2016 | V (0/7) | No | No | No | No | No | No | No |

| *Conduct Questions |
| --- |
| 1. Were inclusion and exclusion criteria of the study population well described and followed? |
| 2. Was the intervention well described and was there adherence to the intervention assignment? (for 2-group designs, was the control exposure also well described?) Both parts of the question need to be met to score ‘yes’. |
| 3. Were the measures used clearly described, valid and reliable for measuring the outcomes of interest? |
| 4. Was the outcome assessor unaware of the intervention status of the participants (i.e., were the assessors masked)? |
| 5. Did the authors conduct and report appropriate statistical evaluation including power calculations? Both parts of the question need to be met to score ‘yes’. |
| 6. Were dropout/loss to follow-up reported and less than 20%? For 2-group designs, was dropout balanced? |
| 7. Considering the potential within the study design, were appropriate methods for controlling confounding variables and limiting potential biases used? |
